# A novel automated behavioral test battery assessing cognitive rigidity in two genetic mouse models of autism

**DOI:** 10.3389/fnbeh.2014.00140

**Published:** 2014-04-29

**Authors:** Alicja Puścian, Szymon Łęski, Tomasz Górkiewicz, Ksenia Meyza, Hans-Peter Lipp, Ewelina Knapska

**Affiliations:** ^1^Department of Neurophysiology, Nencki Institute of Experimental BiologyWarsaw, Poland; ^2^Division of Functional Neuroanatomy, Institute of Anatomy, University of ZurichZurich, Switzerland; ^3^Department of Physiology, School of Laboratory Medicine, Kwazulu-Natal UniversityDurban, South Africa

**Keywords:** autism, perseveration, cognitive rigidity, valproate mouse model, IntelliCages, automatic behavioral tests

## Abstract

Repetitive behaviors are a key feature of many pervasive developmental disorders, such as autism. As a heterogeneous group of symptoms, repetitive behaviors are conceptualized into two main subgroups: sensory/motor (lower-order) and cognitive rigidity (higher-order). Although lower-order repetitive behaviors are measured in mouse models in several paradigms, so far there have been no high-throughput tests directly measuring cognitive rigidity. We describe a novel approach for monitoring repetitive behaviors during reversal learning in mice in the automated IntelliCage system. During the reward-motivated place preference reversal learning, designed to assess cognitive abilities of mice, visits to the previously rewarded places were recorded to measure cognitive flexibility. Thereafter, emotional flexibility was assessed by measuring conditioned fear extinction. Additionally, to look for neuronal correlates of cognitive impairments, we measured CA3-CA1 hippocampal long term potentiation (LTP). To standardize the designed tests we used C57BL/6 and BALB/c mice, representing two genetic backgrounds, for induction of autism by prenatal exposure to the sodium valproate. We found impairments of place learning related to perseveration and no LTP impairments in C57BL/6 valproate-treated mice. In contrast, BALB/c valproate-treated mice displayed severe deficits of place learning not associated with perseverative behaviors and accompanied by hippocampal LTP impairments. Alterations of cognitive flexibility observed in C57BL/6 valproate-treated mice were related to neither restricted exploration pattern nor to emotional flexibility. Altogether, we showed that the designed tests of cognitive performance and perseverative behaviors are efficient and highly replicable. Moreover, the results suggest that genetic background is crucial for the behavioral effects of prenatal valproate treatment.

## Introduction

Along with impairments of social interactions and communication, the most characteristic symptoms of autism are the repetitive behaviors (American Psychiatric Association, 2013). Restricted, repetitive patterns of behavior, interests or activities manifested as, e.g., resistance to change learned response, result in cognitive rigidity (Lopez et al., 2005). Impairments of learning that requires modification of existing behavior, e.g., in a spatial-reversal task, have been reported in autistic children (Coldren and Halloran, 2003). In mouse models of autism this deficit is mirrored by impaired reversal learning in the Morris water maze and T-maze tasks (Moy et al., 2008; Silverman et al., 2010; Guariglia and Chadman, 2013). On the other hand, autism is also diagnosed with accompanying intellectual impairment (American Psychiatric Association, 2013), with cognitive deficits unrelated to repetitive or restricted behaviors. Hence, it remains unclear whether reversal learning impairment that accompanies autism is caused by increased perseveration or by specific cognitive deficits, as the available behavioral tasks do not usually allow for a simultaneous assessment of perseveration and cognitive performance. Moreover, these tasks are carried out on socially isolated animals and require animal handling by an experimenter. These two factors may exert confounding anxiety-related effects on the data obtained from such tests and cause between-laboratory variation (Crabbe et al., 1999). Therefore, we aimed at standardization of a battery of behavioral measures relevant to the repetitive behaviors and reversal learning in the fully automated high-throughput IntelliCage system (Endo et al., 2011; Kobayashi et al., 2013).

The conditions of the tasks were optimized with the use of a mouse model of autism induced by prenatal exposure to sodium valproate (VPA, Wagner et al., 2006; Yochum et al., 2008; Roullet et al., 2010). VPA is an anti-convulsant and a mood stabilizer, as well as a potent histone deacetylase inhibitor (Phiel et al., 2001). It has been shown that exposure to VPA *in utero* is associated with birth defects, cognitive deficits, and increased risk of autism both in humans and rodents. *In utero* VPA treatment constitutes a model with construct and face validity for autism (Roullet et al., 2013). The valproate mouse model, well-characterized in ‘conventional’ behavioral procedures, allowed for comparison between the known phenotypic trait differences and the results obtained in the designed automated tests (Wagner et al., 2006; Yochum et al., 2008; Moldrich et al., 2013; Roullet et al., 2013). In order to assess the effect of genetic background on the results of prenatal exposure to valproate in the current study, we compared C57BL/6 and BALB/c strains of mice. Both strains were previously reported to exhibit prenatal-valproate-exposure-related behavioral impairments (Wagner et al., 2006; Fucic et al., 2010; Gandal et al., 2010; Roullet et al., 2010; Moldrich et al., 2013). Moreover, they are known for their diverse, innate tendency to exhibit stereotypic behaviors (Moy et al., 2008), their diverse sociability (Chen et al., 2009) and social motivation (Kennedy et al., 2012). They were, however, never compared directly or in automated tests allowing for simultaneous assessment of perseveration and cognitive performance. We aimed at comparing two measures of behavioral flexibility, namely place preference reversal learning and extinction of conditioned fear in the valproate-treated mice. To further test the neuronal mechanisms behind cognitive impairments observed in animals prenatally exposed to valproate, we measured CA3-CA1 hippocampal long term potentiation (LTP), whose involvement in hippocampal learning has been shown earlier (Habib et al., 2013).

## Materials and methods

### Subjects

The animals were treated in accordance with the ethical standards of European (directive no. 2010/63/UE) and Polish regulations. All experimental procedures were approved by the Local Ethics Committee. The mice of two strains, C57BL/6J (F18) and BALB/cCrl (F25), were obtained from Mossakowski Medical Research Center, Warsaw and bred in the Animal House of Nencki Institute of Experimental Biology, Warsaw. To assess the effects of prenatal exposure to valproate, animals of different genetic backgrounds were subjected to either saline (SAL) or valproic acid (VPA) prenatal exposure (600 mg/kg on E13, for more details see Supplementary). The animals were group (in experiments carried out in the IntelliCages) or single housed (for fear conditioning experiments) and maintained at a 12 h/12 h light/dark cycle with water and food provided *ad libitum*. In housing and experimental rooms temperature was adjusted to a stable level of 23–25°C, with humidity levels between 10–25%.

The IntelliCage experiments were performed on 36 C57BL/6 (3 cohorts) and 42 BALB/c (4 cohorts) prenatally VPA-treated male mice, 9 C57BL/6 (1 cohort) and 7 BALB/c (1 cohort) prenatally SAL-treated males, as well as 32 C57BL/6 (3 cohorts) and 22 BALB/c (2 cohorts) control (non-treated) male mice, whereas for fear conditioning training we used 20 C57BL/6J (2 cohorts) and 25 BALB/c (2 cohorts) prenatally VPA-treated male mice and 29 C57BL/6 (3 cohorts) and 17 BALB/c (2 cohorts) control male mice. All fear conditioned mice were earlier subjected to the IntelliCage training. Following the IntelliCage training they were separated and thereafter single housed. For the extracellular recordings of *in vitro* LTP 8 control and 7 VPA-treated C57BL/6 male mice, 7 control and 7 VPA-treated BALB/c male mice were used. The animals exploited in LTP recordings were experimentally naïve. Since the behavioral performance of SAL-treated mice and non-treated control mice did not differ, their results were merged in the presented analyses.

In order to reduce aggression in BALB/c group housed males we enriched pre-experimental environment and utilized rat-sized cages allowing for lowering of territorial behaviors. Moreover, overtly aggressive BALB/c males were removed from the group cages and were not used in further procedures.

### Training in the IntelliCages

At least 3 weeks before the behavioral tests male mice were housed together, in the same groups as during the subsequent experimental procedures. To individually identify animals in the IntelliCage system all mice were subcutaneously injected with glass-covered microtransponders (11.5 mm length, 2.2 mm diameter; Trovan, ID-100) under isoflurane anesthesia. Microtransponders emit a unique animal identification code when activated by a magnetic field of the IntelliCage antennas. After transpondering procedure, the subjects were moved from the housing facilities to the experimental rooms and adapted to the shifted light-dark (LD) cycle for 4 to 10 days (the dark phase shifted from 20:00–8:00 to 13:00–01:00 or 12:00–24:00 accordingly to the summer/winter time).

#### Apparatus

Behavioral tests were performed in the IntelliCage, a fully automated, computer controlled system, which can be used for long-term monitoring of behavior of group-housed mice (NewBehavior AG; http://www.newbehavior.com). The plastic cage (size 55 × 37.5 × 20.5 cm) was equipped with four operant learning chambers, which fit into the corners of the housing cage. Access into the chamber was provided via a tube, with a built-in transponder codes reader (antenna). This design restricted access to the learning chamber only for a single mouse at the time. The chamber, equipped with a proximity sensor, contained two openings permitting access to drinking bottles. Poking a nose into the openings (nosepoke response) activated an infrared beam-break response detector. An automatically operated door controlled access to liquid. In addition, the cage contained a sleeping shelter in the center, on the top of which the animals could climb to reach the food located in the feeder in the lid of the cage (food *ad libitum*). Each visit to the operant chamber, as well as each nosepoke and the amount of water consumed (number and duration of licks) was recorded for each individual animal. The cage control unit would permit the access to particular bottles according to preprogrammed schedules, depending on the assignment of the mice to different test groups within the same cage. The system ran continuously for 13 days. During that time behavioral activity of the mice was monitored from the experimenter's office *via* intranet. Except for the technical breaks and cage exchange (once a week), the mice were not disturbed. The light intensity was carefully adjusted in every cage (Supplementary Figure S1).

#### Behavioral training

Several cohorts consisting of 10–12 mice (see Subjects) were subjected to the 13-day IntelliCage protocol, divided into four phases: simple adaptation, nosepoke adaptation, place preference learning, and reversal learning, see Table 1.

**Table 1:**
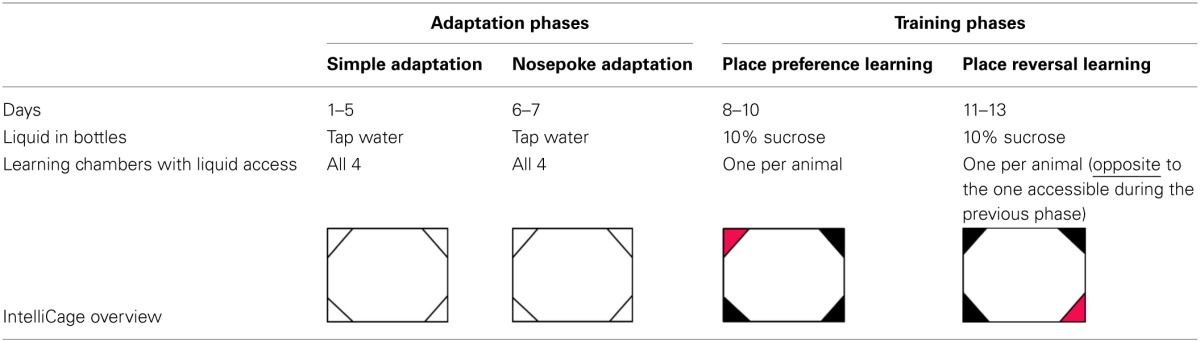
**Scheme of the behavioral experiments**.

*Mice were subjected to the experimental procedures that lasted 13 days. Adaptation phase was followed by the place and reversal learning protocols; white, tap water access, black, no access to bottles, pink, 10% sucrose solution access. During the training phases no more than 3 mice could drink from the same conditioning unit. During all phases of the training visits and nosepokes were registered in all four corners of the cage*.

***Adaptation phase.*** During Simple Adaptation phase, all doors were open and access to water was unrestricted (5 days). During the Nosepoke Adaptation phase, all doors were closed and opened only when an animal poked a nose (nosepoke response) into one of the two openings placed inside each corner. When an animal removed the spout from the opening, the door closed automatically. During the adaptation phase each of 8 bottles contained tap water.

**Figure 1:**
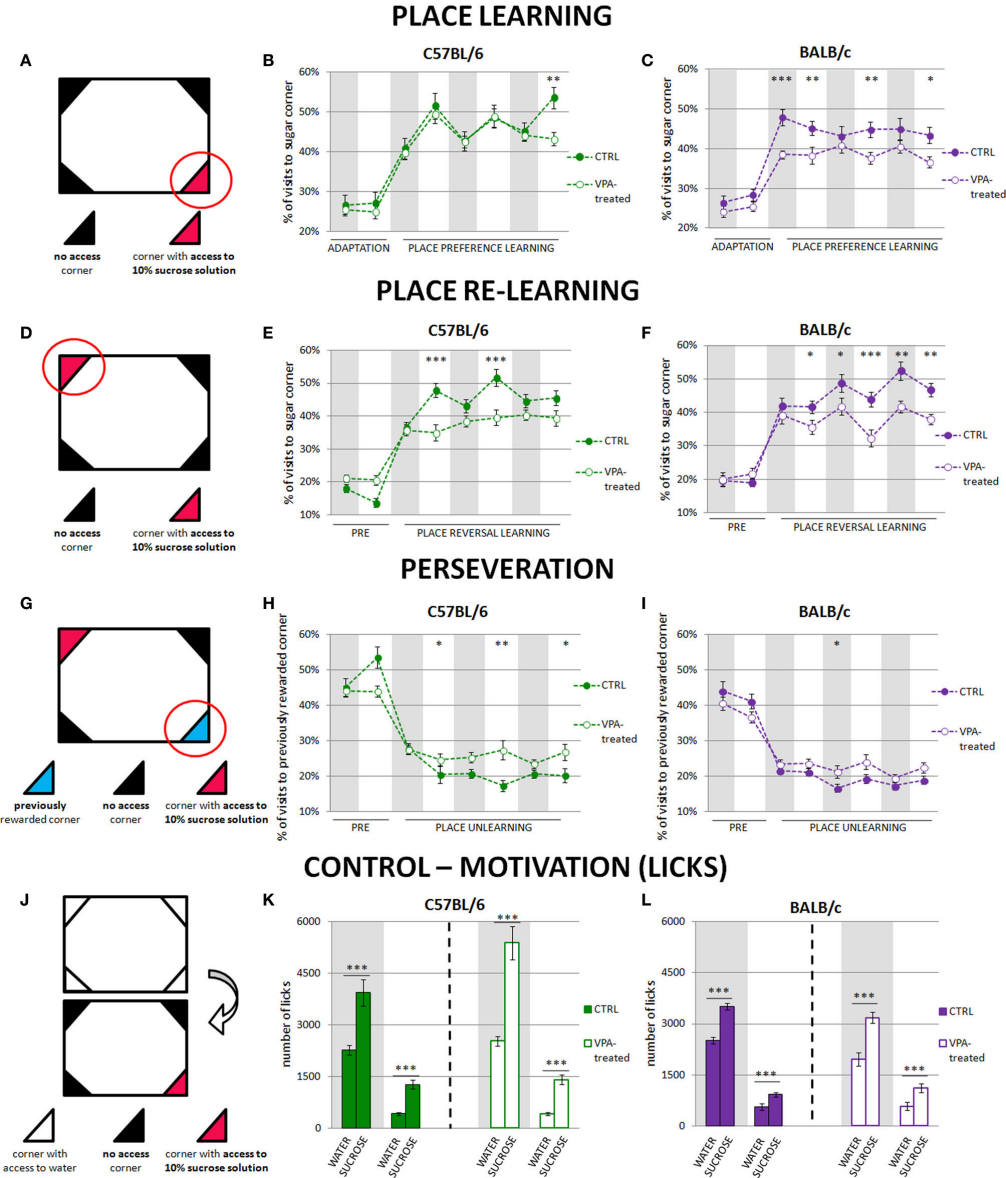
**Impairment of reward motivated place learning is much stronger in valproate-treated BALB/c than C57BL/6 mice**. Place learning deficits in valproate-treated C57BL/6 mice are related to perseverative behaviors. The graphs show percentage of correct visits (in the corner with sweetened water). The results are presented separately for the day and night phases, and gray shaded areas represent the periods of the experiment when the lights were off. **(A)** Initial position of reward (sucrose-sweetened water). **(B)** Valproate-treated C57BL/6 mice (*n* = 36) acquired place preference at the similar level as the control animals (*n* = 41). **(C)** Valproate-treated BALB/c mice (*n* = 42) were severely impaired in reward motivated place learning as compared to the control mice (*n* = 29). **(D)** Reversal of reward position to the opposite corner. **(E)** C57BL/6 valproate-treated mice (*n* = 36) showed fewer correct visits during the light phases of the light-dark cycle than the control animals (*n* = 30). **(F)** BALB/c valproate-treated mice (*n* = 42) showed fewer correct visits during the whole training comparing to the control mice (*n* = 29). **(G)** Visits to the previously rewarded corner provide a measure of perseveration. **(H)** Valproate-treated C57BL/6 mice (*n* = 36) visited the previously rewarded corner more often than the control animals (*n* = 30) during the light phases of the light-dark cycle. **(I)** There were no differences between valproate-treated (*n* = 42) and control (*n* = 29) BALB/c mice in visiting no longer rewarded corner. **(J)** Motivation control—comparison of the amount of 10% sucrose solution and tap water drunk by valproate-treated and control animals. The amount of water was measured during the last dark and last light phases of nosepoke adaptation, whereas amount of 10% sucrose solution—during the first dark and first light phase of the place preference learning. Valproate-treated and control animals of both strains drank significantly more sucrose than water during both dark and light phases of the experiment **(K,L)**. Dots represent the actual data, while dashed lines serve to guide the eye. Error bars represent s.e.m. *p < 0.05, **p < 0.01, ***p < 0.001 (Mann-Whitney *U*-Test for comparisons of two independent groups: C57BL/6 or BALB/c control vs. valproate-treated mice).

***Place preference learning.*** During this phase access to the drinking bottles was restricted to only one of the IntelliCage learning chambers for each mouse. The learning chamber with water access was assigned randomly, with no more than 3 mice drinking from the same conditioning unit. Such procedure minimizes social modulation of learning (Kiryk et al., 2011). To increase animals' motivation for visiting the assigned corner, tap water was replaced by 10% sucrose solution (Sigma-Aldrich), which is strongly preferred by both investigated mice strains (Figures 1K,L).

***Place reversal learning.*** After 3 days of place preference learning, the location of the learning chamber rewarded with sweetened water was changed, to the opposite corner of the cage. All other conditions of the experiment were the same as during the place preference learning.

### Fear conditioning protocol

Valproate-treated and control mice of C57BL/6 and BALB/c strains were submitted to 4 phases of training: fear conditioning, extinction, extinction testing and renewal testing. For fear conditioning (Knapska and Maren, 2009), mice were placed in the conditioning chambers (MedAssociates) in context A, allowed 3 min free exploration, and then received five tone (CS, 20 s; 85 dB; 5 kHz)-footshock (US, 1 s; 0.6 mA) pairings (CS+US) with 60-s intertrial intervals (ITIs). 30 s after the final shock animals were returned to their home cages. For 5 subsequent days animals were submitted to extinction protocol in context B, where 12 tones with no paired foot-shocks were presented (20 s; 85 dB; 5 kHz, 60-s ITI). On the last day mice were tested in both contexts, B (in the morning) and A (in the afternoon). Testing consisted of two 20-s tone CS presentations (85 dB; 5 kHz; 60-s ITI). Fear response to the CS tone during the conditioning, extinction and test phases, was assessed by measuring freezing behavior. Freezing behavior was recorded using a camera placed in front of each chamber. Digital video was saved on and automatically analyzed by VideoFreeze software (MedAssociates system) installed on a computer system located in an adjoining room. Freezing was only scored if the mouse was immobile for at least 1 s. For each session, the freezing observations were transformed to a percentage of total observations. Freezing response was measured in 20-s period following the CS's presentation.

### Behavioral data analysis

The numbers of visits, nosepokes, and tube licks were analyzed for individual mice in 12-h time bins. Additional, more detailed analyses in 6-h instead of 12-h periods were also performed. However, since the 6-h periods within the light and dark phases of the light-dark cycle did not differ in any consistent way we presented the data in 12-h bins. During the Place preference and Place reversal learning phases the percentage of correct responses (number of visits or nosepokes in the corner with liquid access to number of visits or nosepokes made in all four conditioning units of the experimental cage) was calculated. To test perseverative behaviors during the place reversal training, percentages of visits and nosepokes in the previously rewarded corner (rewarded during the place preference training) were calculated. Since the visiting and nosepoking patterns were very similar, we decided to show only one of the measures (visits) in the results section. Moreover, number of tube-lickings of the bottles containing sweetened and tap water was analyzed to establish reward value of 10% sucrose solution.

### Pioneer software package for IntelliCage data screening and analysis

The behavioral data from the IntelliCage system were analyzed using custom software package written in Python programming language (Python 2.7 with NumPy and SciPy libraries). First, raw data files generated by the IntelliCage system were loaded, merged, and tested to identify data segments corrupted due to hardware malfunctions; corrupted segments (if present) were excluded from further analysis. The data excluded from this analysis were: 60 h to 96 h of place preference learning in one C57BL/6 control cohort, 24 h to 48 h of reversal learning and perseveration in one C57BL/6 valproate-treated cohort, 72 h to 96 h of reversal learning and perseveration in one BALB/c control cohort, 60 h to 96 h of place preference learning in one BALB/c valproate-treated cohort; also, due to technical problems one of the experiments carried out on C57BL/6 non-treated animals after place preference learning phase had to be terminated and no data from reversal learning phase were acquired for this cohort. The lack of proper data saving did not interfere with experimental protocol and had no impact on the experimental procedures. Independently of data assembling, the animals were subjected to the stable, preprogrammed conditions. For that reason it is possible to analyze latter data (e.g., from place reversal learning phase), even if the former data (e.g., from place preference learning phase) are missing or corrupted. The relevant quantities (numbers and cumulative durations of visits in specific corners, numbers of nosepokes and tube-lickings) were calculated in 12-h time bins and saved in spreadsheets for statistical analysis and plotting.

### *In vitro* LTP recordings

For extracellular recordings 2.5–to 3.5-month-old male mice were used. Animals were anaesthetized with isoflurane and decapitated. The brains were instantly removed and placed in cold artificial cerebrospinal fluid (aCSF: NaCl 117 mM, MgSO_4_ 1.2 mM, KCl 4.7 mM, CaCl_2_ 2.5 mM, NaHCO_3_ 25 mM, NaH_2_PO_4_ 1.2 mM, 10 mM glucose, bubbled with carbogen) and both hemispheres were cut into 400 μm coronal slices with a vibratome (LeicaVT1000S). Slices containing hippocampus were placed in a recording interface chamber (Harvard Apparatus) to recover for at least 1.5 h before the start of recordings. The slices were continuously perfused with carbogenated CSF at 33°C. Field excitatory postsynaptic potentials (fEPSPs) were recorded using a glass pipette filled with 20 mMNaCl (impendence 1.0–3.0 MΩ) from the stratum radiatum in CA1 area of the hippocampus. To evoke fEPSP, Schafer collateral-commissural afferents were stimulated every 30 s (test pulses at 0.033 Hz, 0.1 ms) with bipolar metal electrodes (FHC, USA). The intensity of test stimulus was adjusted to obtain fEPSP with slopes of one-third of the maximal response. After at least 15 min. of stable baseline, LTP was induced tetanicaly (three trains of 100 Hz, 1 s stimulation, separated by 3 min). After the end of the tetanic stimulation, a test pulse was subsequently applied for at least 90 min. Recordings were amplified (EX4-400 Dagan Corporation, USA), digitized (POWER1401, CED, UK) and slopes of fEPSP were analyzed on-line and off-line. For analysis of LTP, the response slopes were expressed as a percentage of the average response slopes during the baseline period prior to LTP induction.

### Statistical analysis

Statistical analysis of behavioral and electrophysiological data was performed with Statistica 8.0 software (StatSoft). None of the datasets from the behavioral experiments met the criteria for parametric analyses, so these data were subjected to Mann-Whitney *U*-Test for comparison of two independent groups. LTP results were analyzed with repeated-measures ANOVA. The criterion for statistical significance was the probability level *p* < 0.05.

## Results

### Impairment of reward motivated place learning is much stronger in valproate-treated BALB/c than C57BL/6 mice

Cognitive performance of C57BL/6 and BALB/c valproate-treated mice was assessed during place preference and place reversal learning. In the place preference test, the mice were supposed to learn that sweetened water was accessible by nose-poking in only one of the four corners within the large cage (i.e., the “correct” corner, Figure 1A). The level of performance of the C57BL/6 valproate-treated mice and their respective controls was similar (Figure 1B). In contrast, the BALB/c valproate-treated mice did not reach the performance level of the control mice throughout the entire training (Figure 1C). Moreover, comparison of C57BL/6 and BALB/c control mice showed that though the level of performance of BALB/c mice was higher during the first 12-h phase of the place preference learning, in the subsequent light phases of the light-dark cycle in the place preference training C57BL/6 made more visits to the rewarded corner (Figure S2A).

After 3 days of the place preference learning, the reward location was changed and sweetened water was accessible in the opposite corner (place reversal learning paradigm, Figure 1D). C57BL/6 valproate-treated mice made significantly fewer correct visits (to the corner with sweetened water) than the control mice; however this difference was visible only during the light phases of the light-dark cycle in the training (Figure 1E). In contrast, the level of performance of BALB/c valproate-treated mice was lower throughout the entire training as compared to their respective controls (Figure 1F). Moreover, comparison of C57BL/6 and BALB/c control mice revealed differences in the performance level related to light-dark cycle similar to those observed during place preference learning (Figure S2B).

To test whether the observed impairment of reward motivated place learning could be attributed to either reduced thirst or impaired taste discrimination we counted the number of licks on the bottles containing water (during the adaptation phase) or sucrose solution (during the training) (Figure 1J). Both valproate-treated and non-treated animals clearly distinguished between the tap and sweetened water and highly preferred the latter (Figures 1K,L): to see the comparison of liquids consumption for control animals of both tested strains (see Figure S2D). As differences in general activity could potentially influence the obtained results, we compared the number of visits to all corners in the valproate-treated and control mice during the learning phases. Throughout the place preference learning C57BL/6 control subjects made (average ± s.e.m. in dark/light phases of the dark-light cycle) 439 ± 48/123 ± 15 visits, while valproate-treated C57BL/6 animals made 472 ± 15/109 ± 5 visits. BALB/c control mice made 578 ± 71/174 ± 19 visits, whereas valproate-treated BALB/c animals made 469 ± 31/132 ± 11 visits. In the course of place reversal learning C57BL/6 control subjects made 486 ± 43 /159 ± 25 visits, while valproate-treated C57BL/6 animals made: 452 ± 22/98 ± 8 visits. BALB/c control mice made 431 ± 32/158 ± 14 visits, whereas valproate-treated BALB/c animals made 533 ± 36/145 ± 11 visits. The lack of significant differences between the valproate-treated and control mice (with one exception of light phase of the place preference learning in VPA-treated and control BALB/c mice) in the overall number of visits disfavors non-learning explanations of the observed results (e.g., impaired motivation). Moreover, to test for sensory/motor repetitive behaviors, we compared the percentage of visits in four conditioning units within the cage during the adaptation period (Figure S3). Neither C57BL/6 nor BALB/c valproate-treated mice presented restricted exploration pattern.

### Valproate-treated C57BL/6 mice but not valproate-treated BALB/c mice show perseverative behaviors

Since cognitive impairments observed in valproate-treated mice may result either from perseverative behaviors or other specific cognitive impairments (e.g., spatial learning deficits), we assessed perseveration during rewarded place learning. To this end, the percentage of visits to the previously rewarded corner (the corner rewarded during the place preference learning phase of the training) was measured during place reversal learning (Figure 1G). Valproate-treated C57BL/6 mice visited previously rewarded operant chamber more often than the control animals during all light phases of the light-dark cycle in place reversal training (Figure 1H). In contrast, even though they were severely impaired in place preference and reversal learning, valproate-treated BALB/c mice do not show perseverative propensity for repetition of previously learned behavioral patterns (Figure 1I). C57BL/6 control mice visited the previously rewarded corner more often than BALB/c mice at the beginning of the training (Figures 1H,I, Figure S2C). Interestingly, impaired re-learning (of a new position of the rewarded corner) exhibited by the valproate-treated C57BL/6 mice in the light phase was concurrent with a larger percentage of visits to the previously rewarded corner (Figures 1E,H). The same relationship was seen in the more detailed analysis performed in 6-h instead of 12-h periods (Figure S4). This behavior can be interpreted as perseverative. Moreover, it may at least partly explain why the valproate-treated C57BL/6 mice could not achieve better results during re-learning phase.

### Impairment of place preference learning is associated with reduced hippocampal long-term potentiation (LTP)

**Figure 2:**
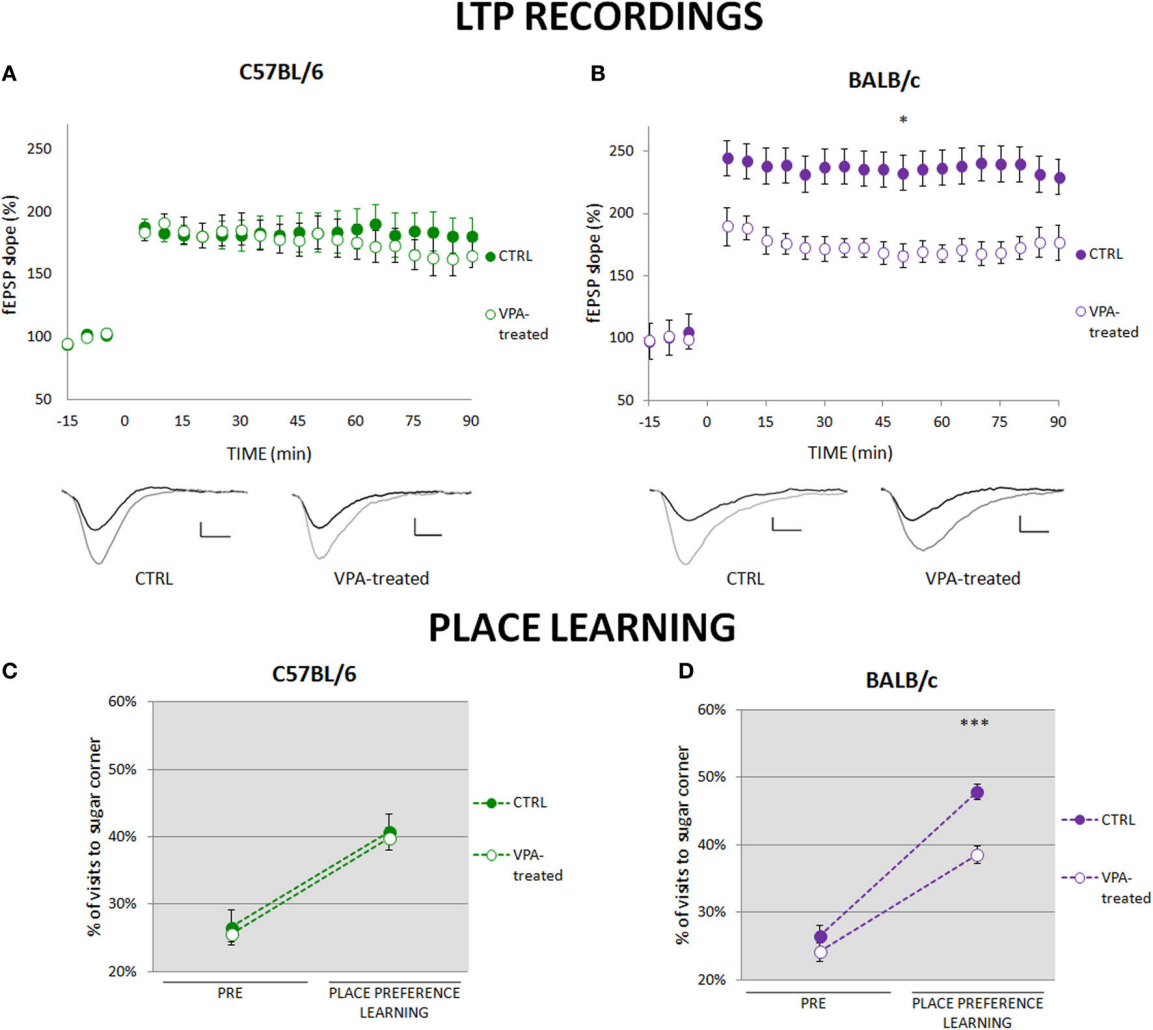
**Impairment of place preference learning is associated with reduced hippocampal long-term potentiation (LTP)**. **(A)** LTP in the CA1 hippocampal pathway was similar in valproate-treated (*n* = 7) and control (*n* = 8) C57BL/6 mice. **(B)** LTP was significantly reduced in valproate-treated BALB/c mice (*n* = 7) as compared to the control animals (*n* = 7; repeated measures ANOVA: *F*_(1, 12)_ = 8.19 *p* < 0.05). **(C)** Valproate-treated (*n* = 36) and control (*n* = 41) C57BL/6 mice acquired place preference at the same level. **(D)** Valproate-treated BALB/c mice (*n* = 42) showed impaired place preference learning comparing to the control animals (*n* = 29; Mann-Whitney *U*-Test comparisons of two independent groups). Dots represent the actual data, while dashed lines serve to guide the eye. Error bars represent s.e.m. ^*^p < 0.05, ^***^p < 0.001.

To investigate synaptic plasticity that may underlie place learning we compared long-term potentiation (LTP) in the CA3-CA1 pathway. In the C57BL/6 valproate-treated mice and their controls LTP was induced at similar levels (Figure 2A), whereas in the BALB/c valproate-treated mice LTP was significantly reduced as compared to the control animals (Figure 2B). Moreover, comparison of C57BL/6 and BALB/c control mice showed that LTP is induced at significantly higher level in BALB/c mice (Figures 2A,C, Figure S5). The observed between-strains differences correspond to the performance in place preference learning task in the first phase of the training (see Figures 2C,D, Figure S5).

### Automated behavioral tests of cognitive and perseverative behaviors are highly replicable

**Figure 3:**
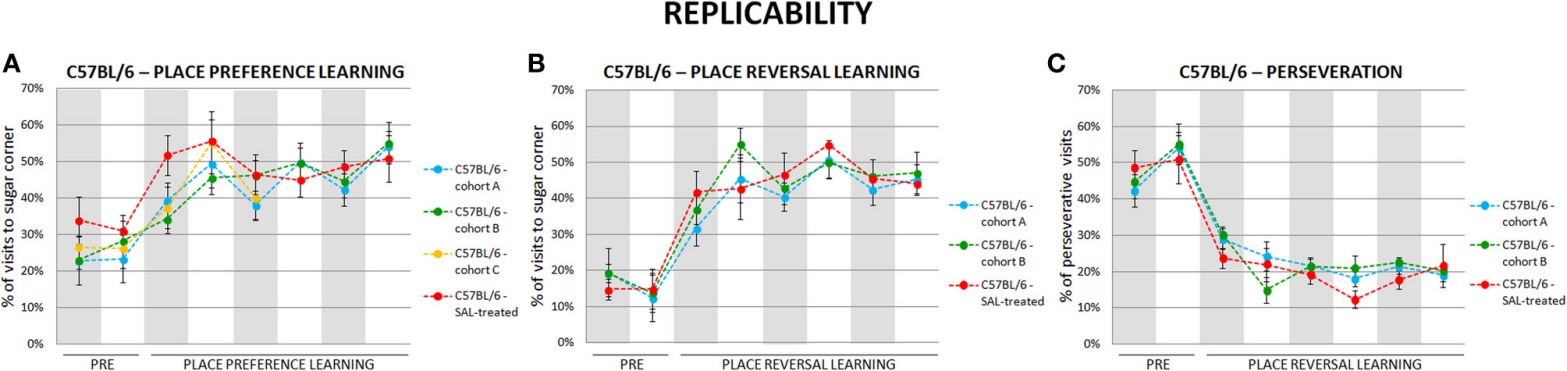
**Self-designed automated behavioral tests are highly replicable**. The results of place preference learning **(A)** in four cohorts (*n* = 41), reversal learning **(B)** and perseveration **(C)** in three cohorts (*n* = 30) of C57BL/6 mice. Dots represent the actual data, while dashed lines serve to guide the eye.

To test replicability of the presented behavioral measures we performed each behavioral test in several cohorts of valproate-treated and control C57BL/6 and BALB/c mice. The results were highly replicable (as an example see Figure 3).

### Fear conditioned valproate-treated C57BL/6 mice but not BALB/c mice exhibit lower level of freezing during fear extinction and decreased contextual fear renewal

**Figure 4:**
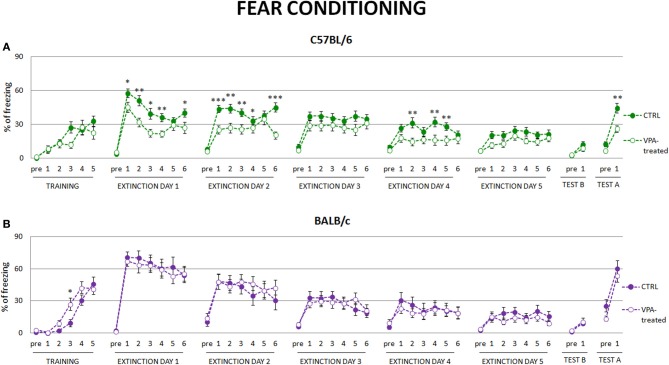
**Fear conditioned valproate-treated C57BL/6 mice but not BALB/c mice exhibit lower level of freezing during fear extinction and decreased contextual fear renewal**. **(A)** During extinction session valproate-treated C57BL/6 mice (*n* = 20) decreased freezing response to the conditioned stimulus to a larger degree than the control animals, as well as showed decreased fear renewal to the conditioning context. **(B)** Such differences were not observed in valproate-treated BALB/c mice. Dots represent the actual data, while dashed lines serve to guide the eye. Error bars represent s.e.m. *p < 0.05, **p < 0.01, ***p < 0.001 (Mann-Whitney *U*-Test for comparisons of two independent groups: C57BL/6 or BALB/c control vs. valproate-treated mice).

As extinction of conditioned fear is considered to be one of the measures of behavioral flexibility we investigated this behavior in valproate-treated mice of both strains. We observed that valproate-treated C57BL/6 mice expressed less freezing to the conditioned stimuli in subsequent fear extinction sessions, suggesting that they extinguished fear memories to a larger degree than the controls. They also showed reduced fear renewal, when introduced to the context in which they were conditioned (see Figure 4A). Neither of those effects was observed in valproate-treated BALB/c mice (see Figure 4B).

## Discussion

In the present study, we standardized a battery of automated tests that allow for testing of cognitive abilities along with perseverative behaviors in group-housed mice. We show that our measures of cognitive performance and perseverative behaviors are highly replicable. By applying the designed tests, we were able to document impairments of place learning related to perseveration in C57BL/6 valproate-treated mice. In contrast, BALB/c valproate-treated mice displayed severe deficits of place learning not associated with perseverative behaviors. Cognitive rigidity observed in C57BL/6 valproate-treated mice was not accompanied by restricted exploration pattern. Furthermore, we showed that the cognitive deficit of C57BL/6 valproate-treated mice was modulated by the circadian rhythm, as it was prominent only during the light phase of the day. We also observed impairments of long-term potentiation (LTP) at CA3-CA1 synapses in the hippocampus that paralleled the performance in the IntelliCage place preference test, but not reversal learning or perseveration. Finally, our data indicate, that reversal learning and extinction of conditioned fear, considered as measures of behavioral flexibility, are differently affected in valproate-treated mice. C57BL/6 valproate-treated mice extinguished conditioned fear to a larger degree than the control animals, whereas in BALB/c valproate-treated mice such differences were not observed.

It is well known that social reinforcements have little impact on behavior of autistic individuals (Schultz et al., 2000; Sepeta et al., 2012). Yet, there is also a growing body of evidence for altered non-social reward perception (Dichter et al., 2012). For instance, recent studies in humans using reward-motivated learning have shown that autistic patients care less about information of reward contingencies. Also, performance in the reward-motivated tasks was related to repetitive behavior symptoms (Damiano et al., 2012). Some authors suggest that restricted interests and intensified attachment to characteristic objects may result from enhanced reward value and its impact on patients behavior (Sasson et al., 2008; Dichter et al., 2012). In order to delineate the role of repetitiveness on cognitive function in reward-based paradigms we developed a setting targeted to reflect particular behavioral malfunctions in autistic individuals.

To optimize the conditions of the behavioral tasks described here we used a mouse model of autism induced by prenatal exposure to sodium valproate, which has been previously shown to cause social behavior deficits in C57BL/6 (Gandal et al., 2010) and BALB/c (Yochum et al., 2008) mice. The valproate-treated BALB/c mice were also significantly impaired during place learning in the Morris water maze (Crawley, 2012). Valproate-treated C57BL/6 mice have not been tested for spatial learning performance so far, but it has been shown that they are often engaged in intense repetitive self-grooming behavior (Gandal et al., 2010; Moldrich et al., 2013). Our data are consistent with the results showing significant impairment of spatial learning in valproate-treated BALB/c mice. In our tests, we also observed repetitive behaviors in valproate-treated C57BL/6 mice.

We showed that valproate-treated mice of C57BL/6 and BALB/c strains were differently influenced by the light-dark cycle. C57BL/6 valproate-treated mice, in contrast to BALB/c valproate-treated mice, showed performance impairments limited to the light phases of the reversal learning. We hypothesize that the light phase is an inactive period of light-dark cycle, in which visits to the operant chambers are less frequent and separated by longer intervals. Consequently, performance of correct responses (visits or nosepokes) is more difficult and more long-term memory-dependent. In contrast, during the dark phases visits in the conditioning units are more frequent and the chances of finding the correct corner are increased. The difference observed between the C57BL/6 and BALB/c strains may arise from slight differences in their circadian melatonin production dynamics. BALB/c mice display a very short but high peak of pineal melatonin concentration in the middle of the dark period, while C57BL/6 mice show smaller, but prolonged (two broader peaks) release of melatonin from pineal gland after the middle of the dark period (Vivien-Roels et al., 1998). Since sodium valproate treatment was found to significantly decrease the sensitivity of melatonin to light (Hallam et al., 2005), a diverse impairment of circadian activity of these mice could be expected. In prenatally valproate-treated rats abnormal circadian activity rhythm was accompanied by increased frontal cortex serotonin release (serotonin is a precursor for melatonin synthesis, Tsujino et al., 2007). Such mechanism may in turn exacerbate differences in melatonin synthesis in our models. The current findings emphasize the role of circadian rhythms in the development and severity of autism spectrum disorder. Autistic children are known to have lower daytime and nighttime levels of 6-sulphatoxymelatonin, which correlate with severity of their language and social interaction impairments and the repetitive use of objects (for review see Tordjman et al., 2013). Their performance is also correlated with high excretion rate of cortisol and several other circadian corticosteroids (Lakshmi Priya et al., 2013).

In the present study we observed that genetic background significantly affected the results of prenatal exposure to sodium valproate. Despite the same treatment and experimental conditions, cognitive functioning of C57BL/6 and BALB/c valproate-treated mice was different. Valproate-treated BALB/c mice displayed severely impaired place learning, i.e., deficits in reward motivated place and reversal leaning, but no perseverative tendencies. Electrophysiological data are consistent with the behavioral findings, as valproate-treated BALB/c mice had significantly deteriorated LTP in the CA1 field of the hippocampus. On the other hand, valproate-treated C57BL/6 mice showed no generalized cognitive impairments in place preference learning and no CA1 LTP decrease; however they exhibited very specific deficits in relearning of reward location, which were accompanied by persistent visits to the previously reinforced locations.

The observed genetic background-related differences in response to VPA challenge may result from differences in prenatal development pace of C57BL/6 and BALB/c strains, diverse metabolism of the drug or its interaction with genes activated at certain point of the development. Further studies are required to elucidate the underlying mechanisms. At this point, our results show that perseverative impairments, most relevant for mouse models of autism, were observed only in C57BL/6 valproate-treated mice, which makes it a more valuable animal model for this set of autism-like symptoms.

Earlier studies have shown that hippocampal LTP (Moser et al., 1998), especially along the CA3-CA1 pathway (Habib et al., 2013), is involved in spatial learning measured in the Morris water maze. The Morris water maze is one of the behavioral tasks most often used to assess cognitive abilities of mice. In our previous study we observed similar spatial memory deficits in mice tested in the water maze and place preference paradigm in the IntelliCage system (Kiryk et al., 2011). Therefore, we decided to further investigate this relation by measuring LTP in the CA1 field of the hippocampus in C57BL/6 and BALB/c valproate-treated and control mice and assess its correlation with place preference learning in the IntelliCage system. Our data suggest that similar mechanisms of neuronal plasticity may be involved during spatial learning in the Morris water maze and place preference learning in the IntelliCage.

Comparing reversal learning and extinction of conditioned fear, two measures of behavioral flexibility, we found that they were differently affected by valproate-treatment in C57BL/6 and BALB/c mice. C57BL/6 valproate-treated mice displayed specific deficits in reversal learning associated with perseverative behaviors, whereas they extinguished conditioned fear to a larger degree than the control animals. Our study shows that this effect depends on genetic background, as BALB/c valproate-treated mice showed significant impairment of place learning, whereas their conditioned fear extinction was as efficient as in the control mice. Altogether the current data suggest that reversal learning and fear extinction, reflecting, respectively, cognitive and emotional flexibility, involve different mechanisms.

The results of behavioral tests are often difficult to replicate between laboratories, or even within the same laboratory (Wahlsten et al., 2001). Such difficulties are, at least partially, caused by the problems with tests standardization. Our data on test replicability show that we are able to obtain consistent results with several cohorts of experimental and control mice, corroborating the results of the study by Krackow et al. (Codita et al., 2012). It should be stressed that out of many parameters that could be obtained in the IntelliCage system, only those identified as stable and replicable enough were used in the presented analyses. An example of a labile and not replicable parameter, rejected from analyses, was duration of a visit to a given corner. In addition, the IntelliCage system avoids behavioral effects of social isolation and forced human handling stress. Another advantage of testing animals in the IntelliCage system is that it employs the natural repertoire of mouse behaviors, thus enhancing ecological relevance of the designed protocols. Such species-specific testing of mouse behavior allows for assessing impairments of everyday adaptive functioning of mice living in a social group, so it appears more relevant to common deficits observed in autistic patients than most of the changes observed during testing single animals.

In summary, “conventional” mouse tests are prone to artifacts caused by social isolation and handling stress, mimicking autism-like behaviors and reduced cognitive abilities. This is further exacerbated by low inter-test reliability and mouse strain differences. Our battery of behavioral tests in the fully automated and standardized IntelliCage system addresses all these issues as demonstrated by the data sets.

### Conflict of interest statement

The authors declare that the research was conducted in the absence of any commercial or financial relationships that could be construed as a potential conflict of interest.

## Supplementary material

### Sodium valproate prenatal exposure based mouse model of autism

The mice were mated with pregnancy confirmed by the presence of a vaginal plug on embryonic day 0 (E0). On E13, pregnant females received a single subcutaneous injection of 600 mg/kg VPA (Sigma-Aldrich) dissolved in saline (SAL). Control females received an equal volume of SAL only. The drug was dissolved in SAL in the concentration of 58–63 mg per 1 ml. The volume of the injection fluid was <0.35 mL, allowing for a proper absorption of the solution. The litter sizes of the VPA-treated C57BL/6 mice varied from 2 to 12 pups, whereas in BALB/c mice from 4 to 8 pups. Therefore, a cohort (10–12 mice) used in the IntelliCage experiments consisted of males from 3 to 6 litters in both investigated strains. To obtain one cohort we usually mated at least 12–15 females, from which at least 8–9 had the pregnancy confirmed by the presence of a vaginal plug on embryonic day 0. The postnatal mortality of the offspring was higher in BALB/c than in C57BL/6 animals. Also, we had cases of BALB/c mice being born without one of the front limbs, eye or whiskers (not used in the experiments). Moreover, we observed that in BALB/c strain it was more difficult than in C57BL/6 to obtain the sufficient number of pregnancies that eventually led to giving birth. All of the experiments were performed with male 2.5- to 5-month-old offspring.

### Standardization of the illumination level in IntelliCage apparatus

Our pilot data revealed considerable impact of light intensity on the activity of the mice in the IntelliCage apparatus. Therefore in the current study the illumination levels were standardized between all experimental cages used. The light intensity was measured in four points inside of each cage, with the feeding lid and shelter for mice placed to mimic conditions during testing procedures. Every measurement was repeated twice, and the mean value was calculated. Light measurement points are marked in Figure S1. During the experiments light levels were set to 45–60 lux in points 1–2 and to 70–90 lux in points 3–4.

Figure S1 **Lighting intensity adjustment**. **(A)** The IntelliCage apparatus, with four conditioning units in the corners and a feeder located in the middle of the cage. The shelter for mice (red) is located below the feeder. **(B)** Schematic overview of IntelliCage apparatus. The grid located in the middle section of the drawing represents feeder. The numbers in circles mark 4 points where the measuring instrument (Digital light meter, model 5202, Kyoritsu) was placed during the light set-up procedure. We thank Anna Mirgos and Jakub Kowalski for providing us with the IntelliCage photograph.

Figure S2 **Mice of C57BL/6 and BALB/c strains are differently influenced by the light-dark cycle during reward motivated place learning and re-learning. (A)** The level of performance of C57BL/6 (*n* = 41) and BALB/c (*n* = 29) differed during the light-dark phases of the place preference training. **(B)** Performance differences between C57BL/6 (*n* = 30) and BALB/c (*n* = 29) mice during place re-learning as related to the light-dark cycle. **(C)** Though C57BL/6 mice (*n* = 30) visited the previously rewarded corner more often than BALB/c mice (*n* = 29) at the beginning of the training, the different levels of performance in the PRE period makes it difficult to interpret this behavior as perseveration. **(D)** Animals of both investigated strains consumed significantly more 10% sucrose solution than water. Dots represent the actual data, while dashed lines serve to guide the eye. Error bars represent s.e.m. **p* < 0.05, ***p* < 0.01, ****p* < 0.001. (Mann-Whitney *U*-Test for comparisons of two independent groups: C57BL/6 and BALB/c mice).

Figure S3 **Neither C57BL/6 nor BALB/c valproate-treated mice show restricted exploration pattern**. To test for sensory/motor repetitive behaviors we examined the percentage of visits in the most frequently visited conditioning unit throughout adaptation period. For higher ecological accuracy dark **(A,B)** and light **(C,D)** phases of 24 h cycle were analyzed separately. Neither C57BL/6 **(A,C)** nor BALB/c **(B,D)** valproate-treated mice presented stronger tendency for visiting one particular corner than control animals. Circles represent percentage of visits in the most frequently visited conditioning unit for each mouse in all examined cohorts.

Figure S4 **The results of analysis in 6-h time bins**. The description of the figure as in Figure 1. Dots represent the actual data, while dashed lines serve to guide the eye. Error bars represent s.e.m. **p* < 0.05, ***p* < 0.01, ****p* < 0.001 (Mann-Whitney *U*-Test for comparisons of two independent groups: C57BL/6 or BALB/c control vs. valproate-treated mice).

Figure S5 **Hippocampal long-term potentiation (LTP) level corresponds to place preference learning efficiency in C57BL/6 and BALB/c strains. (A)** BALB/c mice (*n* = 7) exhibited stronger LTP than C57BL/6 (*n* = 8; repeated measures ANOVA: *F*_(1, 13)_ = 9.11, *p* < 0.05). **(B)** BALB/c mice (*n* = 29) showed higher conditioned place preference during first 12 h of training as compared to C57BL/6 mice (*n* = 41) (Mann-Whitney *U*-Test for comparisons of two independent groups: C57BL/6 and BALB/c mice). Dots represent the actual data, while dashed lines serve to guide the eye. Error bars represent s.e.m. **p* < 0.05, ***p* < 0.01, ****p* < 0.001.
