# Supplementary figures and images for: A novel automated behavioral test battery assessing cognitive rigidity in two genetic mouse models of autism
Source: Front Behav Neurosci. 2014 Apr 29;8:140. doi: 10.3389/fnbeh.2014.00140 (PMC4010752; doi:10.3389/fnbeh.2014.00140)

# ILLUMINATION MEASUREMENT

A

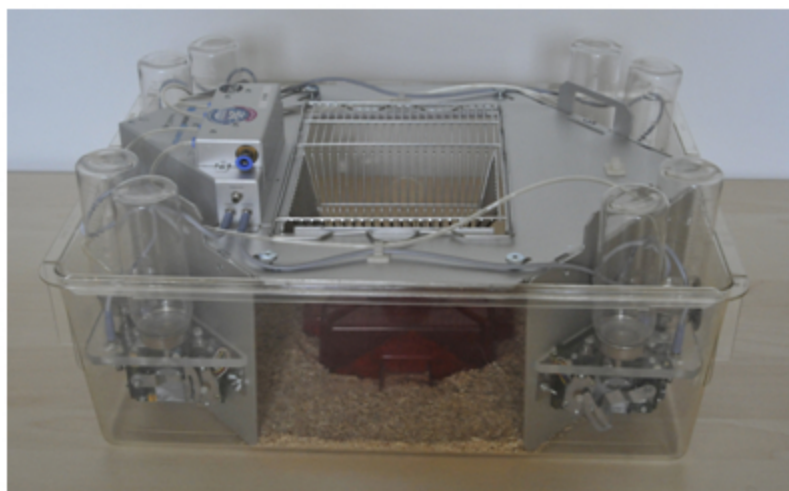

B

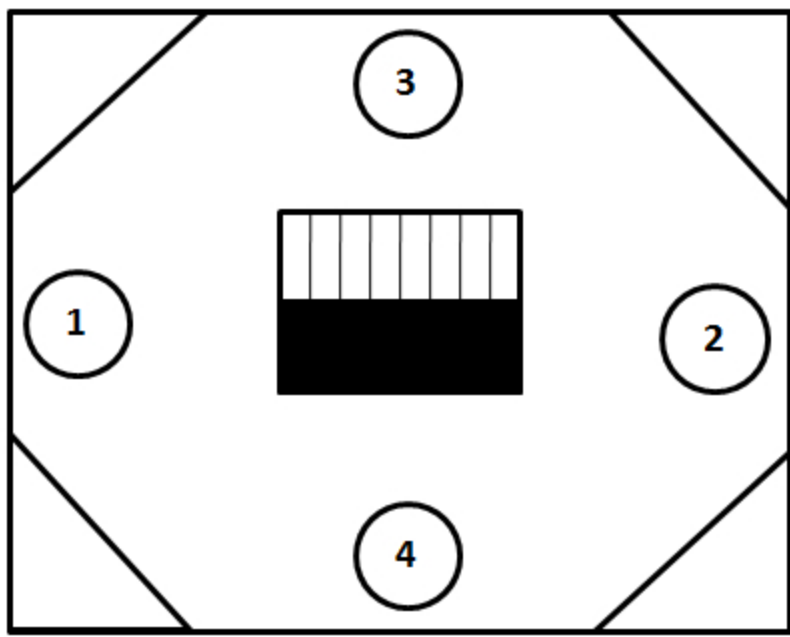

Supplement: Figure S1 — Lighting intensity adjustment. (A) The IntelliCage apparatus, with four conditioning units in the corners and a feeder located in the middle of the cage. The shelter for mice (red) is located below the feeder. (B) Schematic overview of IntelliCage apparatus. The grid located in the middle section of the drawing represents feeder. The numbers in circles mark 4 points where the measuring instrument (Digital light meter, model 5202, Kyoritsu) was placed during the light set-up procedure. We thank Anna Mirgos and Jakub Kowalski for providing us with the IntelliCage photograph. [file Presentation1.PDF]

# PLACE LEARNING

A

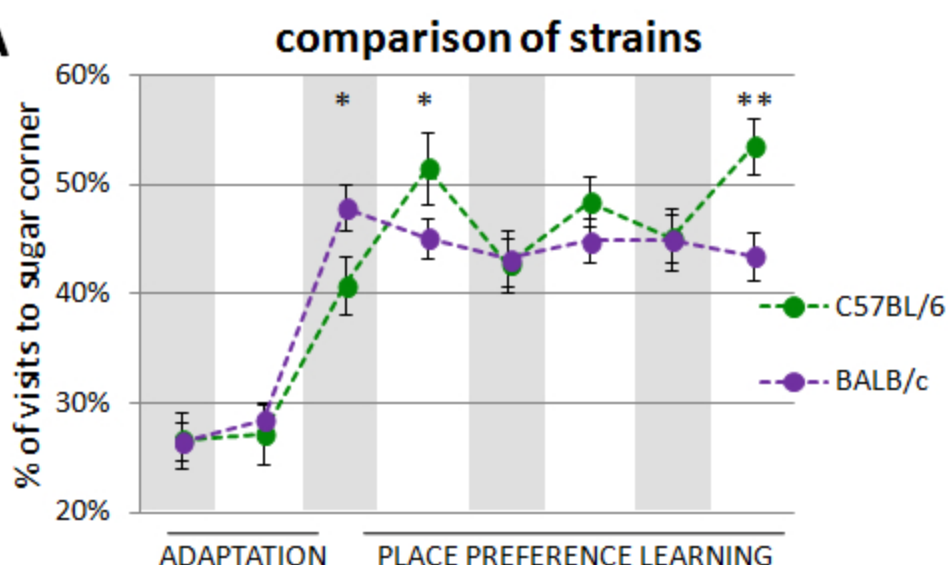

# PLACE RE-LEARNING

B

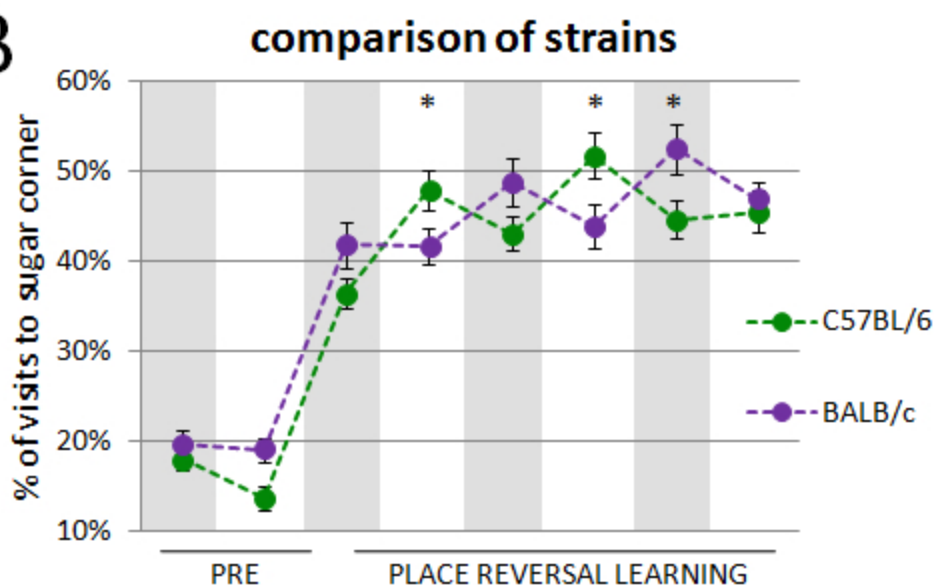

# PERSEVERATION

C

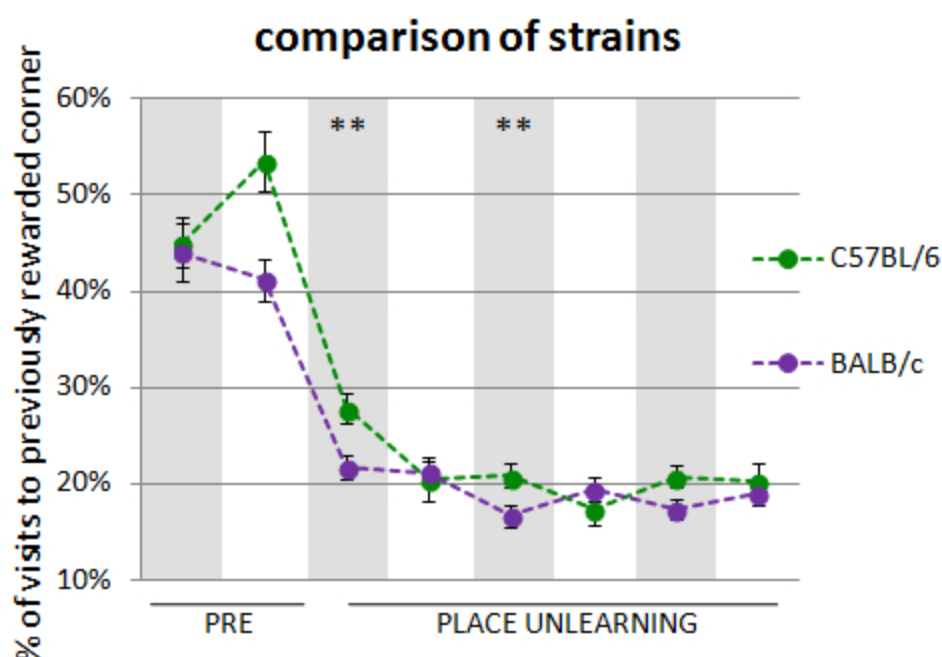

# CONTROL – MOTIVATION (LICKS)

D

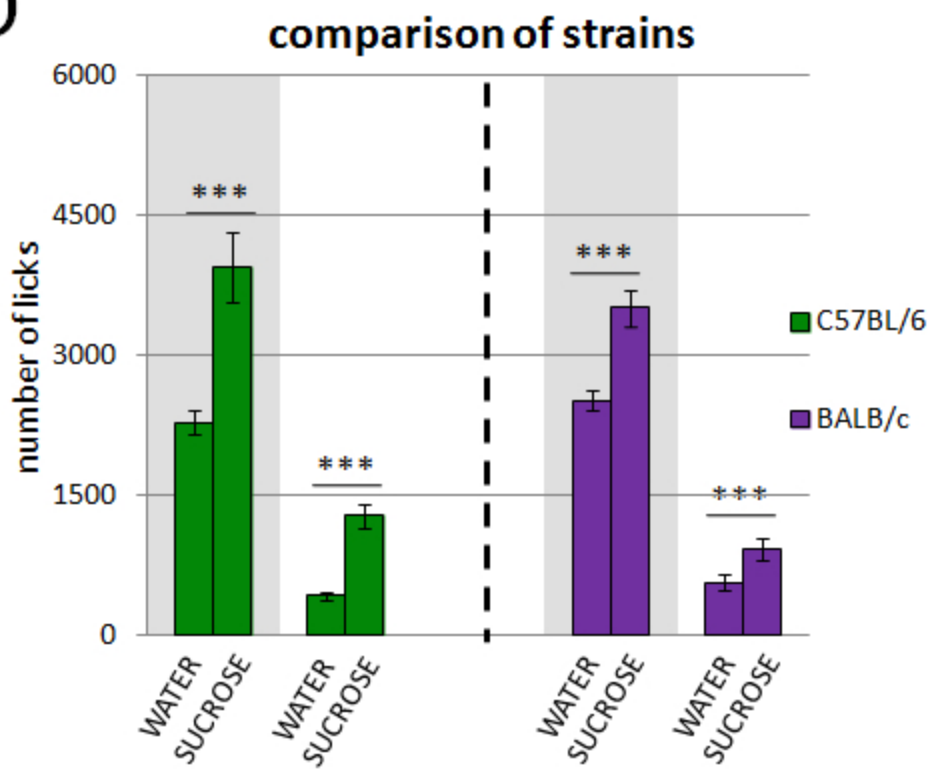

Supplement: Figure S2 — Mice of C57BL/6 and BALB/c strains are differently influenced by the light-dark cycle during reward motivated place learning and re-learning. (A) The level of performance of C57BL/6 (n = 41) and BALB/c (n = 29) differed during the light-dark phases of the place preference training. (B) Performance differences between C57BL/6 (n = 30) and BALB/c (n = 29) mice during place re-learning as related to the light-dark cycle. (C) Though C57BL/6 mice (n = 30) visited the previously rewarded corner more often than BALB/c mice (n = 29) at the beginning of the training, the different levels of performance in the PRE period makes it difficult to interpret this behavior as perseveration. (D) Animals of both investigated strains consumed significantly more 10% sucrose solution than water. Dots represent the actual data, while dashed lines serve to guide the eye. Error bars represent s.e.m. *p < 0.05, **p < 0.01, ***p < 0.001. (Mann-Whitney U-Test for comparisons of two independent groups: C57BL/6 and BALB/c mice). [file Presentation2.PDF]

# CONTROL – SENSORY/MOTOR REPETITIVE BEHAVIORS

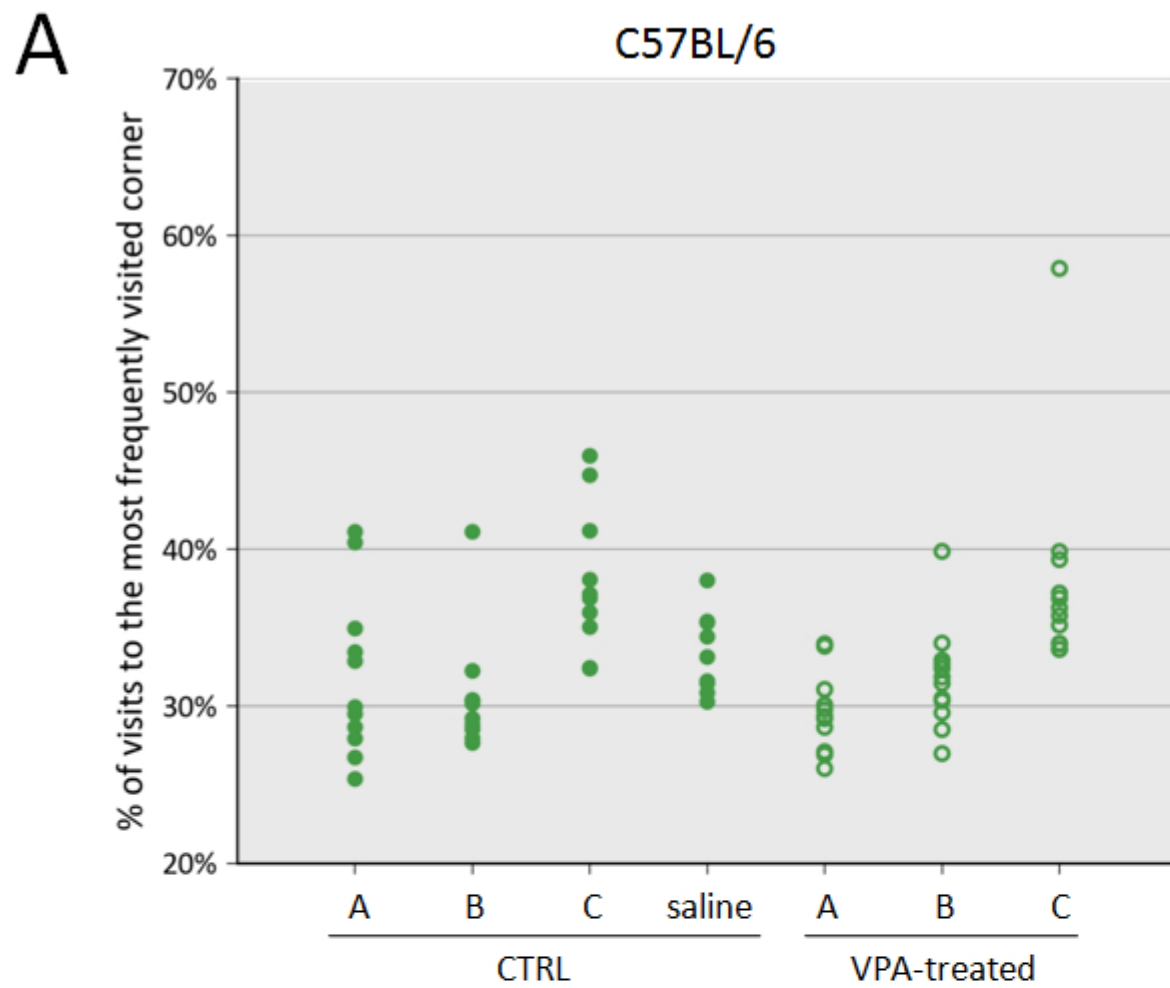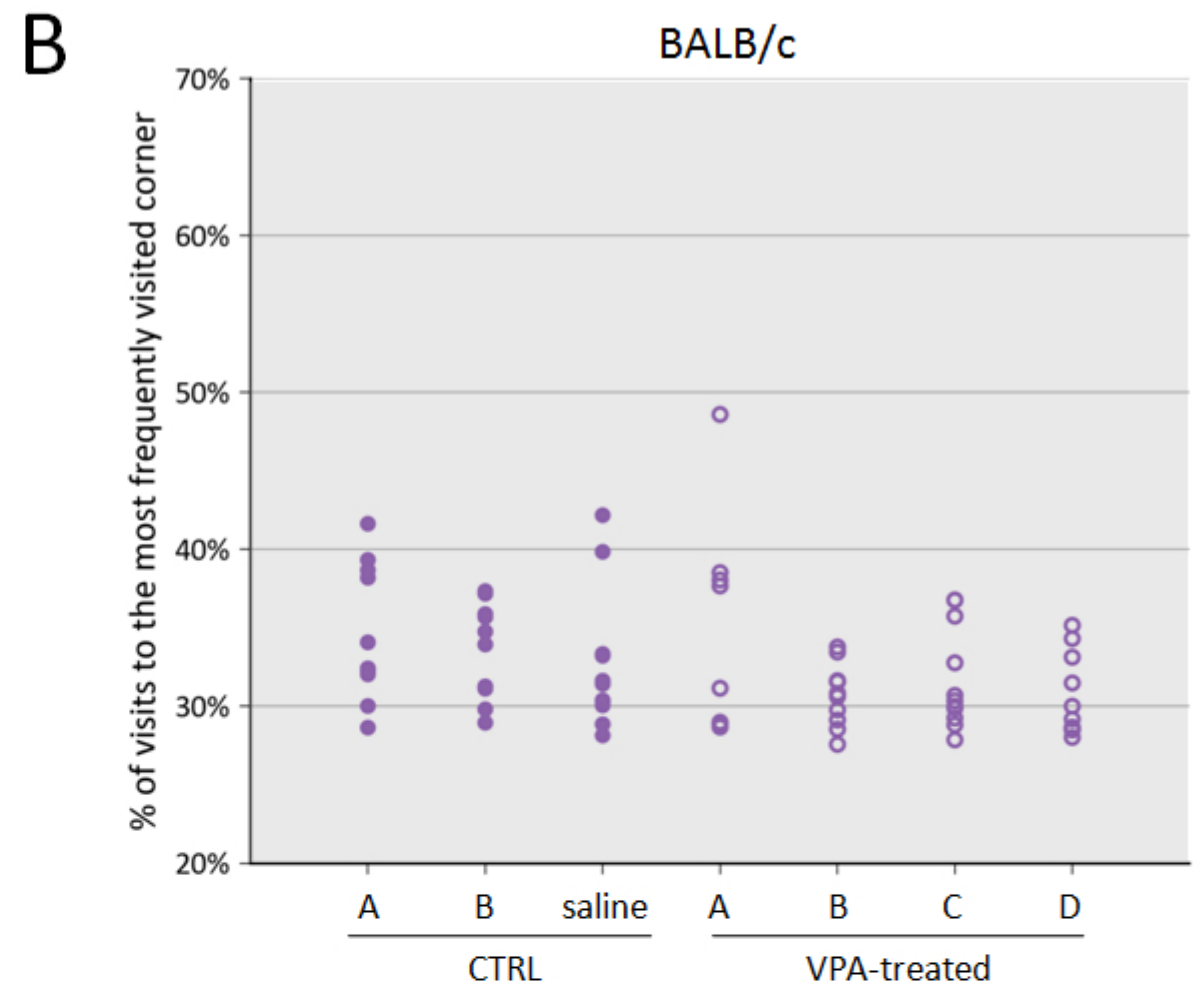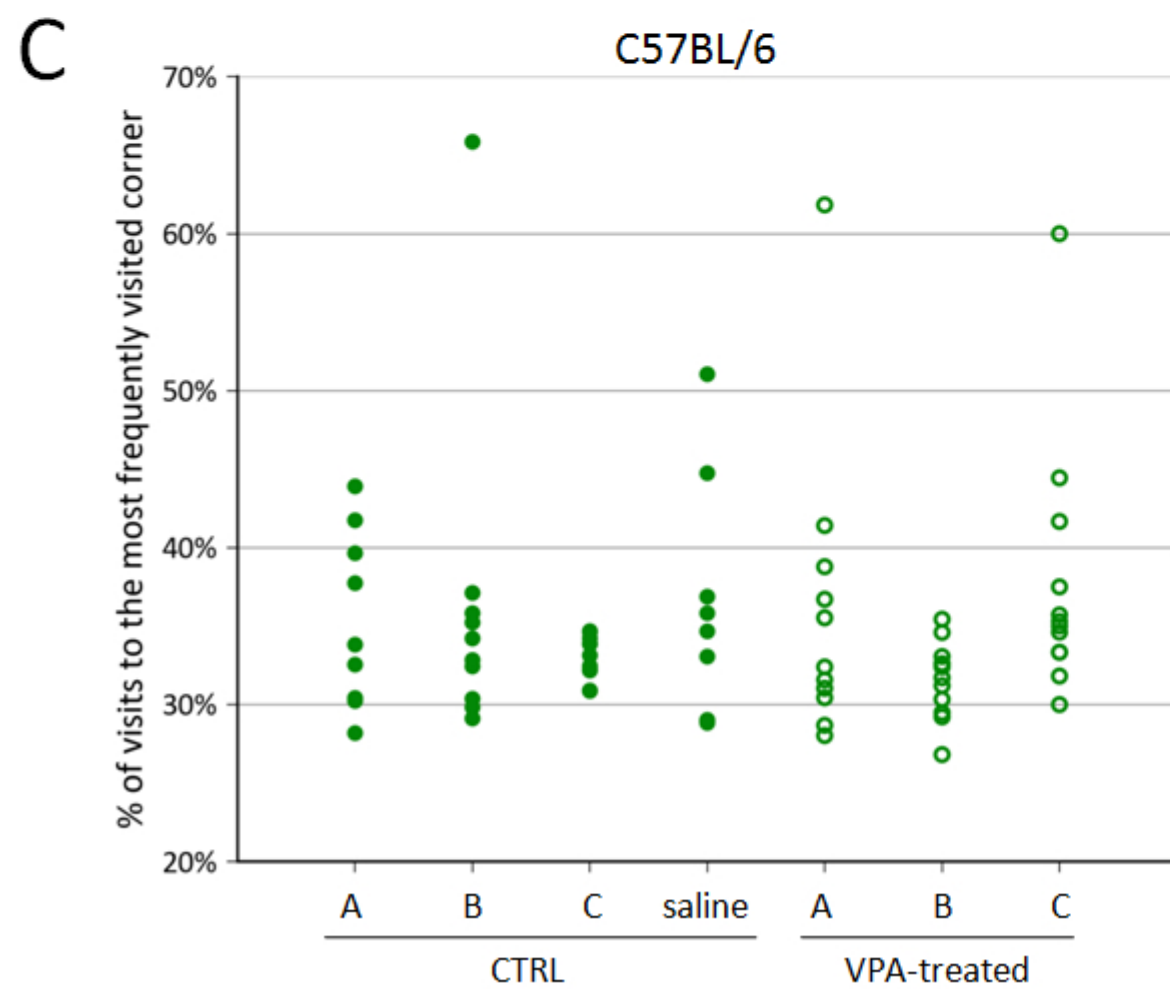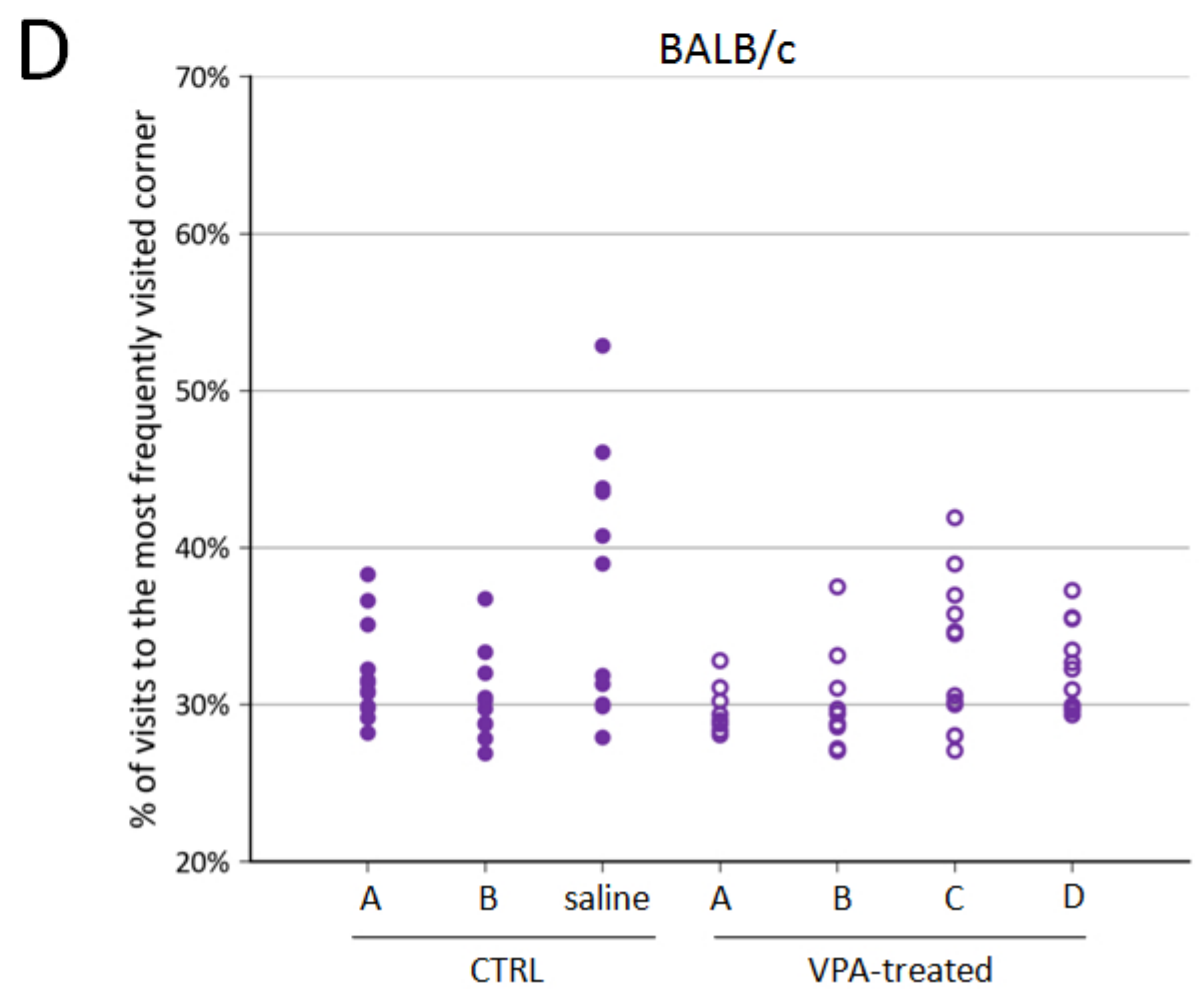

Supplement: Figure S3 — Neither C57BL/6 nor BALB/c valproate-treated mice show restricted exploration pattern. To test for sensory/motor repetitive behaviors we examined the percentage of visits in the most frequently visited conditioning unit throughout adaptation period. For higher ecological accuracy dark (A,B) and light (C,D) phases of 24 h cycle were analyzed separately. Neither C57BL/6 (A,C) nor BALB/c (B,D) valproate-treated mice presented stronger tendency for visiting one particular corner than control animals. Circles represent percentage of visits in the most frequently visited conditioning unit for each mouse in all examined cohorts. [file Presentation3.PDF]

# PLACE LEARNING

**A**

**C57BL/6**

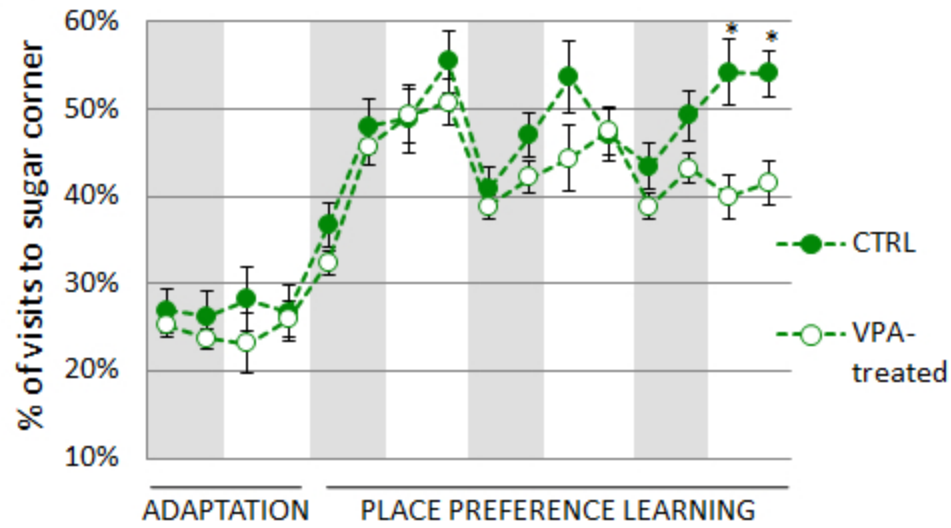

**B**

**BALB/c**

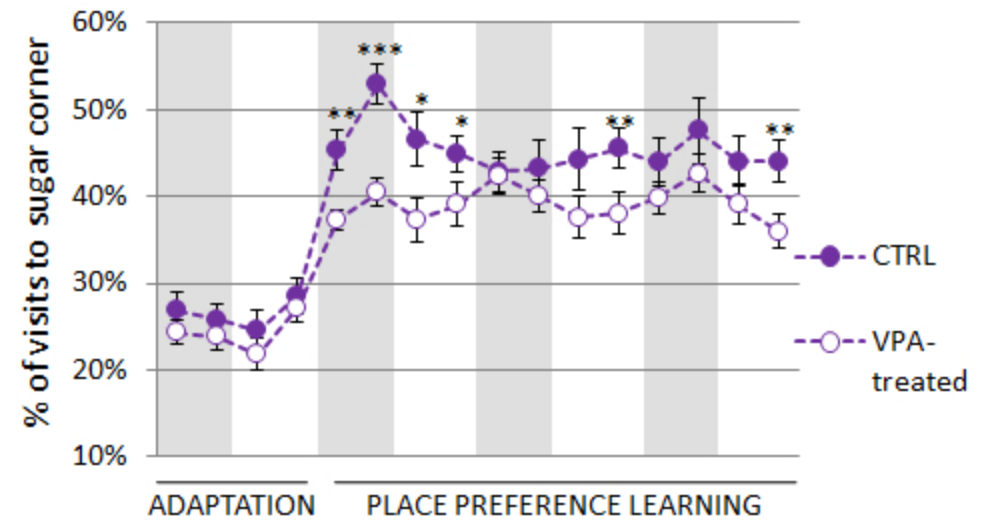

# PLACE RE-LEARNING

**C**

**C57BL/6**

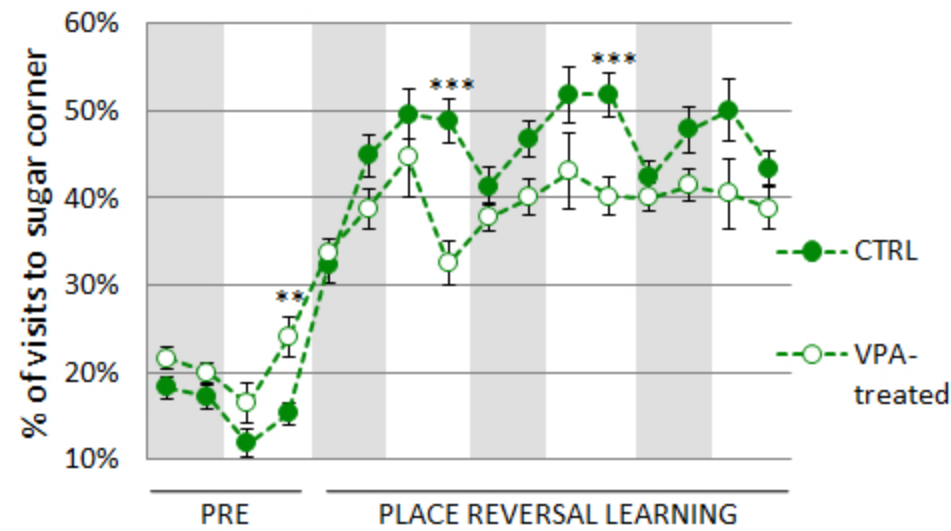

**D**

**BALB/c**

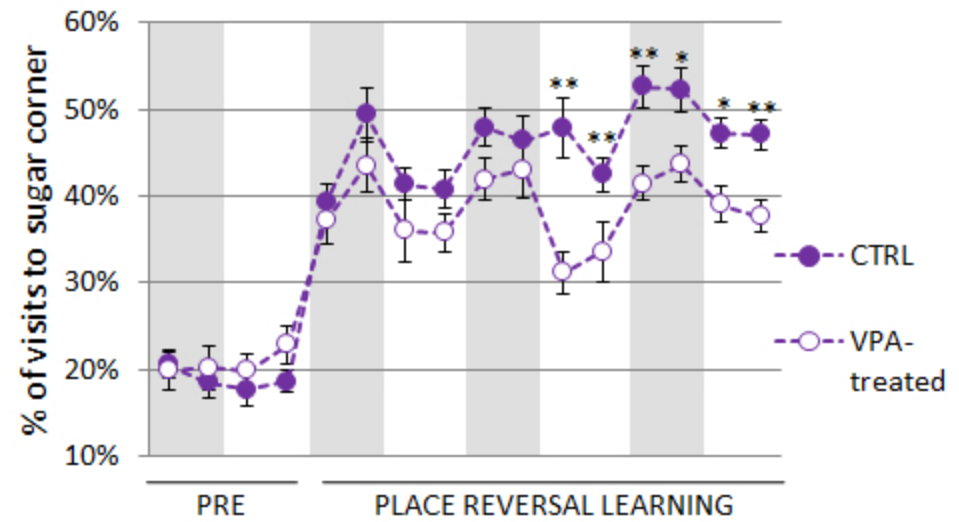

# PERSEVERATION

**E**

**C57BL/6**

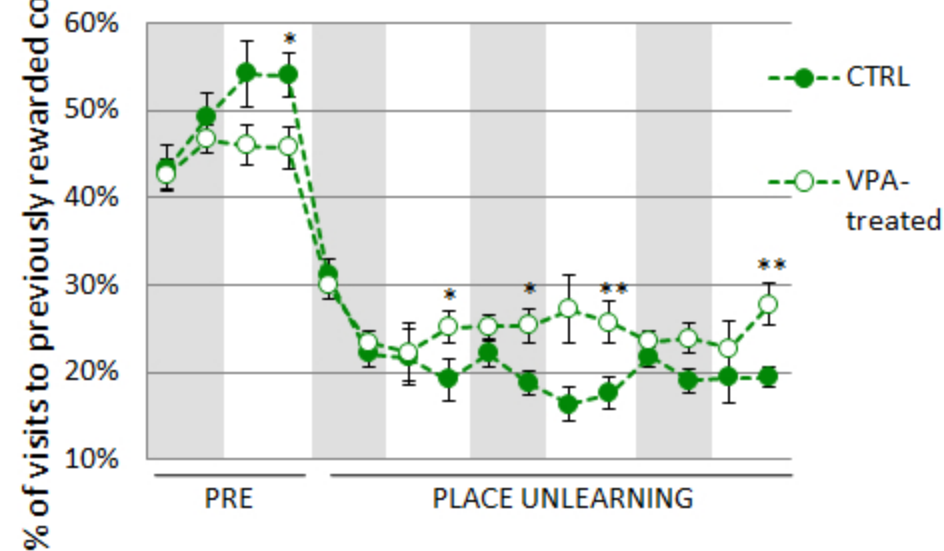

**F**

**BALB/c**

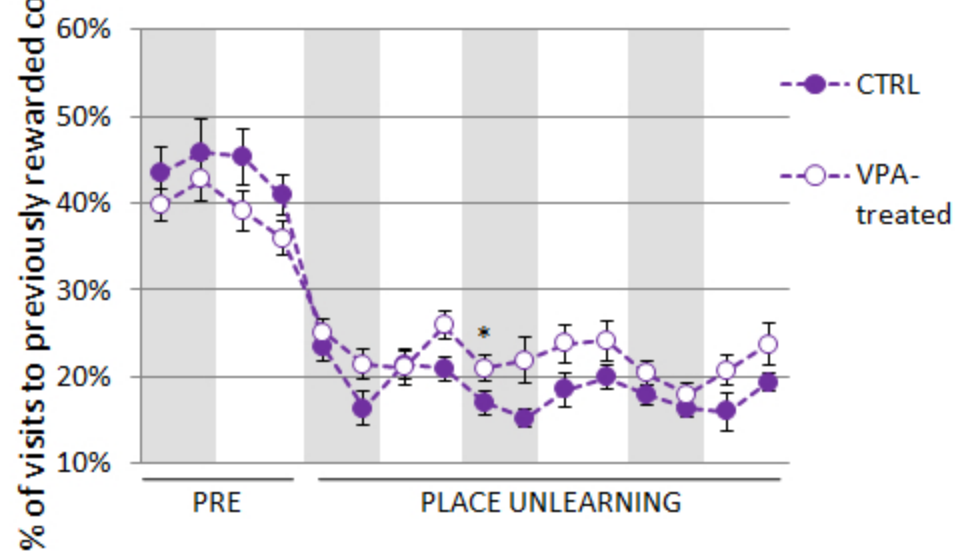

Supplement: Figure S4 — The results of analysis in 6-h time bins. The description of the figure as in Figure 1. Dots represent the actual data, while dashed lines serve to guide the eye. Error bars represent s.e.m. *p < 0.05, **p < 0.01, ***p < 0.001 (Mann-Whitney U-Test for comparisons of two independent groups: C57BL/6 or BALB/c control vs. valproate-treated mice). [file Presentation4.PDF]

# LTP RECORDINGS

A

comparison of strains

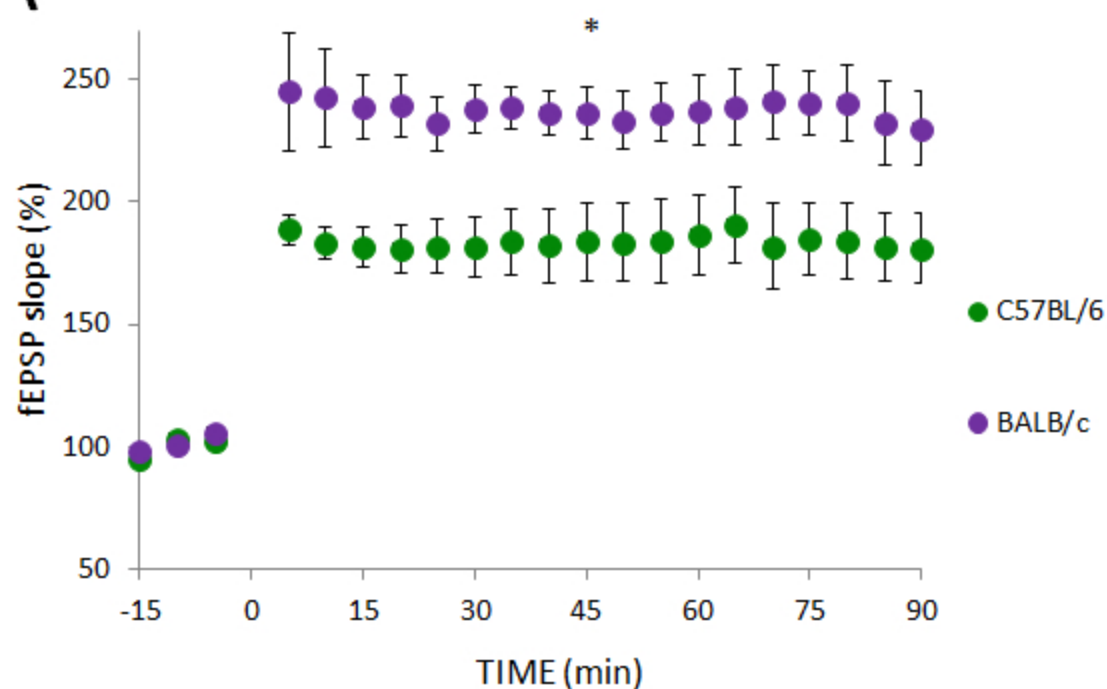

B

comparison of strains

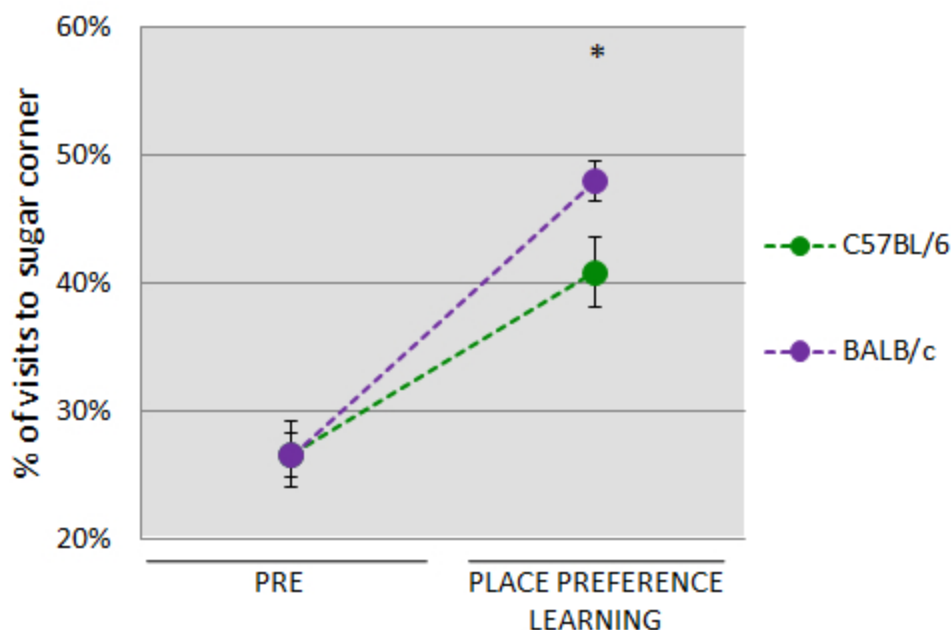

Supplement: Figure S5 — Hippocampal long-term potentiation (LTP) level corresponds to place preference learning efficiency in C57BL/6 and BALB/c strains. (A) BALB/c mice (n = 7) exhibited stronger LTP than C57BL/6 (n = 8; repeated measures ANOVA: F(1, 13) = 9.11, p < 0.05). (B) BALB/c mice (n = 29) showed higher conditioned place preference during first 12 h of training as compared to C57BL/6 mice (n = 41) (Mann-Whitney U-Test for comparisons of two independent groups: C57BL/6 and BALB/c mice). Dots represent the actual data, while dashed lines serve to guide the eye. Error bars represent s.e.m. *p < 0.05, **p < 0.01, ***p < 0.001. [file Presentation5.PDF]
